# Green synthesis of zinc oxide nano particles using *Allium cepa* L. waste peel extracts and its antioxidant and antibacterial activities

**DOI:** 10.1016/j.heliyon.2024.e25430

**Published:** 2024-01-27

**Authors:** Md Faridul Islam, Shariful islam, Md Abdus Satter Miah, A.K. Obidul Huq, Anik Kumar Saha, Zinia Jannat Mou, Md Mahmudul Hassan Mondol, Mohammad Nazrul Islam Bhuiyan

**Affiliations:** a Institute of Food Science and Technology (IFST), Bangladesh Council of Scientific and Industrial Research (BCSIR), Dr. Qudrat-I-Khuda Road, Dhanmondi, Dhaka-1205, Bangladesh; b Department of Food Technology and Nutritional Science, Mawlana Bhashani Science and Technology University, Santosh, Tangail-1902, Bangladesh; c Department of Biochemistry and Molecular Biology, Bangabandhu Sheikh Mujibur Rahman Agricultural University (BSMRAU), Salna, Gazipur-1706, Bangladesh; d Department of Chemical Engineering, Khulna University of Engineering & Technology (KUET), Khulna-9203, Bangladesh

**Keywords:** *Allium cepa* L., Antioxidant, Antimicrobial, Green synthesis, Nanoparticle, Zinc oxide

## Abstract

Synthesis of nanoparticles through the green approach using plant and vegetable extracts has gained popularity since they are thought to be efficient and cost-effective materials. This study is designed to synthesize zinc oxide nanoparticles (ZnO-NPs) from onion waste peel extract (*Allium cepa* L.) via the green synthesis approach. The synthesized ZnO-NPs were characterized by utilizing the UV–Vis spectroscopy, Fourier transform infrared spectroscopy (FTIR), Energy Dispersive X-ray (EDX), Field Emission Scanning Electron Microscopy (FE-SEM) and X-ray Powder Diffraction (XRD)techniques. The nanoparticles formation was confirmed by the UV–Vis sharp absorption spectra at 318 and 322 nm. The synthesized ZnO-NPs size and shape was revealed by the XRD and SEM respectively. Smallest nanoparticle average crystallite size was found 57.38 nm with hexagonal shape. The bioactive functional groups that are in charge of capping and stabilizing the ZnO-NPs was assured by the FTIR data. Further, prepared ZnO-NPs were used to assess their possible antioxidant and antibacterial properties. DPPH test for free radical scavenging showed potential antioxidant properties of the synthesized ZnO-NPs. The antibacterial activity were studied against three clinical strains: *P. aeruginosa*, *E. coli*, and *S. aureus* with the maximum zone of inhibition 13.17 mm, 22.00 mm and 12.35 mm respectively at 100 μg/mL subsequently minimum inhibitory concentration was found 50 μg/mL for *P. aeruginosa*, and *S. aureus* whereas 100 μg/mL for *E. coli*. Antioxidant and antibacterial activity tests appear bio-resource based ZnO-NPs from *Allium cepa* L. extract have effects on free radical and growth of microorganisms.Therefore, it could be a promising candidates for agricultural and food safety applications as an effective antimicrobial agent against pathogenic microorganisms and also can address future biomedical applications after complete *in vivo* study.

## 1 Introduction

Nanotechnology has become a significant branch of research during the last few decades, mostly employed for the evaluation and production of substances at the nanoscale, which typically falls between 1 and 100 nm in at least one dimension [1]. The high surface-to-volume ratio of nanoparticles enhanced the physical characteristics and also increased the reactivity of the nanoparticles. Now-a-days, nanotechnology is referred to as a wonder of modern medicine, since, this field has advanced to the point that new directions in nanoscience are possible, especially in drug delivery, gene delivery, biosensing, and nanomedicine [[2], [3], [4]]. It is also playing a significant role in the field of agriculture, food processing industries, environmental sciences, and biotechnology [5,6].

Metal and metal oxide (MO) NPs are regarded as the anticipated materials because of their characteristics such as solubility, chemical stability, adhesiveness, and surface plasmon resonance [[7], [8], [9]]. Recently, ZnO, CuO, Ag_2_O, Fe_2_O_3_, CaO, NiO, and MgO NPs have received the most attention in literature as having antibacterial action [[10], [11], [12], [13], [14], [15], [16], [17], [18]]. Among those NPs, ZnO is considered to be a prospective contender in the field of biomedicine, particularly for antibacterial, antioxidant, α-amylase inhibitory activity, biosensing, cell imaging, anti-inflammatory, anti-cancer, and anti-diabetes applications [[19], [20], [21], [22], [23], [24]]. So far, various physical and chemical techniques, including sol-gel, colloidal, thermal, sonochemical, hydrothermal, microwave irradiation, thermal breakdown, and rapid precipitation approach have been reported to synthesize ZnO-NPs [25]. The risk of using hazardous chemicals in various NPs synthesis process restricts them in applications in the domains of biotechnology, medicine, environment, biology, and other industrial applications [26]. Synthesis based on bioresources could be one way to overcome such restrictions since it is eco-friedly, less toxic and easy to adapt. Additionally, using waste materials lowers the energy needs and synthesis costs when compared to physical or chemical synthesis processes, highlighting the effectiveness of "green synthesis" [27,28]. Recently, this approach has been applied for ZnO NPs synthesis to evaluate their antioxidant and α-amylase inhibitory activity [23,24]. In addition, polyol present in the plant component (like leaves, fruits, seeds, peels, pulps, roots, barks, and fruits) extracts as sources of bioactive chemicals can stabilize the metallic NPs by acting as chelating and capping agents during fast production of nanoparticles [29,30]. Preceded study showed that ZnO-NPs from leaf extract of *Melia azedarach and Cassia fistula* exerted antimicrobial activity against two medically significant strains namely *Staphylococcus aureus* (*S. aureus*) and *Escherichia coli* (*E. coli*). The antibacterial activity of ZnO NPs produced from *Ruta graveolens* (L.) was observed against four bacterial strains, specifically *Escherichia coli (E. coli), Pseudomonas aeruginosa (P. aeruginosa), Klebsiella aerogenes (K. aerogenes),* and *Staphylococcus aureus (S. aureus)* [22].

However, *Allium cepa* L. (onion), being a cross-pollinated biennial important spice containing several phytochemicals including phenolics, flavonoids, anthocyanins, and organic acids [31,32]. These polyol compounds make onion an outstanding option for the green synthesis of NPs. The ZnO-NPs using *Allium cepa* L. extract showed anti-inflammatory, anti-hyperlipidemic, and cardiovascular protective effects [33,34]. In a recent investigation, Khamis et al., synthesized onion extracts mediated ZnO NPs at an average crystallite size 8.13 nm having hexagonal wurtzite phase and exhibited greated antibacterial activity than Cefotaxime [35]. Biogenic ZnO NPs from *Allium cepa* extracts also showed potential antihyperlipidemic and cardiovascular protective effects by controlling biochemical parameters linked to lipid metabolism [36]. Another study claimed that *Allium cepa* bulb based ZnO NPs have potential antimicrobial, *in vitro* antioxidant and antidiabetic activity [37]. Most of the researchers uses the edible part of the onion to extract the reducing and capping agents. In Bangladesh, huge amounts of dry waste peel of onion is generated everyday and dumped here and there. These are considered as a garbage and polluted the environment. But, it can be a potential source for the extraction of reducing agent since it contains several types of phytochemical such as polyphenols, flavonoids, tannins, saponins, alkaloids and antioxidants etc. [35]. Therefore, to make dried onion waste peel useable, development of an efficient extraction procedure for the synthesize of biogenic metal oxide nanoparticles is indespensible. In addition, the biological method of producing zinc oxide nanoparticles helps to create environmentally sustainable and biocompatible zinc oxide nanoparticles while also reducing the amount of waste that is produced from onions.

In this research, *Allium cepa* L. (onion) waste peel extract were emploed as a reducing and caping agent to synthesize ZnO-NPs. Different approach such as orbital shaking, microwave, and hot plate heating was applied to optimize the extraction and synthesis method. The optimized methods were then used to synthesize ZnO NPs and UV–Vis, FTIR, FE-SEM, and XRD analysis were employed to characterize the synthesized ZnO NPs. The biogenic synthesized ZnO-NPs were further employed to assess their potential antibacterial and antioxidant activities. The antibacterial activity was investigated following the modified Kirby Bauer disc diffusion method against three clinical strains: *E. coli*, *P. aeruginosa*, and *S. aureus*.

## 2 Materials and methods

### 2.1 Raw materials, chemicals and reagents

Dry onion peel waste (*Allium cepa* L.) were collected from a local market in Dhaka. Zinc acetate (Zn(CH_3_CO_2_).2H_2_O), 2,2-Diphenyl-1-picrylhydrazyl (DPPH), methanol, ethanol, and ascorbic acid were procured from Merck, Research-lab, India; Fine Chem Industries, India; Panreac, EU and Merck, Germany, respectively.

### 2.2 Onion peel extract preparation

Dry waste peels of the onions (*Allium cepa* L.) were collected and washed under running water repetedly to eliminate dust particles. The waste peel were then weighed and properly blended with the help of a food blender (Brand: PHILIPS, Model: Simply Silent, 600W). To get the better results, it is important to extract the reducing agent efficiently. Numerous process variables such as the precursor to bio-source ratio, pH, temperature, reaction duration, kind and concentration of the precursor solution are all the important factors in the green synthesis of zinc oxide nanoparticles. Therefore, optimization of the factors involved in producing ZnO NPs is necessary to depict the relationship between different parameters. Considering all this, the extract was prepared at three different concentrations under three distinct conditions. The sample concentrations are: **Condition A:** (**A1**: 50g blended onion waste peel in 100 mL deionized water, **A2**: 50 g blended onion waste peel in 150 mL deionized water, **A3**: 50 g blended onion waste peel in 200 mL deionized water), **Condition B:** (**B1**: 50 g blended onion waste peel in 100 mL deionized water, **B2**:50 g blended onion waste peel in 150 mL deionized water, **B3**: 50 g blended onion waste peel in 200 mL deionized water), and **Condition C**: (**C1**: 50 g blended onion waste peel in 100 mL deionized water, **C2**:50 g blended onion waste peel in 150 mL deionized water, **C3**: 50 g blended onion waste peel in 200 mL deionized water). Microwave heat treatment with a time interval of 2.0 min was applied on Condition A whereas mechanical shaking using an orbital shaker (Brand: Grant-bio, Model: PSU-10i, England) at 200 rpm for 30 min was applied on Condition B to prepare the extract solution respectively. Samples of condition C were kept for microwave heat treatment with a time interval of 2 min followed by boiling for 15 min on a hot plate to prepare the extract solution from condition C. To filter, Whatman No. 1 paper was used and collected the supernatant. The light pink aqueous solutions were then stored at 4 °C to synthesis ZnO NPs.

### 2.3 Synthesis of ZnO NPs

The synthesis of ZnO-NP were completed by following the modified method outlined by Degefa A. et al. [38]. The 20.0 mL aqueous onion waste peel extract solution was transferred in a 250 mL beaker and positioned above a magnetic stirrer. (Phoenix, Model: RSM-0SH). The temperature and rotation per minute (rpm) was set 65 °C and 450 respectively. Then,100.0 mL of 2 mM zinc acetate solution was was gradually added from a graduated burette. After the addition of zinc acetate, the pH was adjusted to 8.0 ± 0.10 and magnetically stirred until the reaction mixture took on a new hue from light pink to yellowish white. After that, it was set to cool to ambient temperature and stand for 4 h and then centrifuged (DSC, Model: 302SMD, Taiwan) at 7000 rpm for 5 min. The supernatant disposed of and precipitate underwent two to three washings with deionized water and subsequently with ethanol. The resulted residue was dried at 130 °C for 3 h to make it moisture free. The sample was then ground with mortar and pestle into fine powders and calcined for 2 h at 500 °C to get rid of any impurities. The powdered sample was transferred in a properly labeled airtight ziplock bag and stored at room temperature for future analysis.

### 2.4 Characterization

The physicochemical properties of ZnO-NPs produced from *Alliumcepa*L. extract have been examined by employing a range of characterization techniques including FTIR, XRD, SEM, EDX and UV–Vis spectrophotometer. The optical characteristics of the synthesized product were analyzed by utilizing UV–Vis spectrophotometer (Model:Specord 205, Analytic Zena, Germany) in the range of 280–450 nm. XRD was also used to examine the materials' crystallinity (Model:Smart LAB, SE, Rigaku, Japan) equipped with Cu-k_α_ radiation and a wavelength of 1.5406 Å. The functional group responsible for the formation of the nano particle were determined by using FTIR (Model:PerkinElmer, USA) spectroscopy between 600 and 4000 cm^−1^. The FESEM was used to record the NP's morphology (Model: JSM-7610F, JEOL, USA), following their combination with acetone and drying on a glass slide to form a thin coating. The elemental maping of the nanomaterial's were determined using SEM-EDX.

### 2.5 Antioxidant acivity

The antioxidant activity of biosynthesized ZnO NPs was determined using the DPPH method described by D. Das et al. [39]. Two distinct procedures were used in the DPPH scavenging assay; one experiment was time-dependent, while the other experiment was concentration-dependent. In summary, 150 mg of produced nanoparticles were sonicated for 10 min at room temperature in a glass tube containing 3.2 mL of a 100 μM DPPH solution. The absorbance was then measured at 517 nm at intervals of 0, 15, 30, and 45 min.

To evaluate the DPPH scavenging activity at various doses, a solution containing 100 μM DPPH was produced in 99.9 % methanol. The analysis was then carried out by combining 3.0 mL of DPPH solution with 1 mL of sample solution at various doses (50 μg/mL, 100 μg/mL, 200 μg/mL, 250 μg/mL, and 500 μg/mL). This combination was then allowed to incubate for 30 min at ambient temperature in the dark. Ascorbic acid was used as the standard and methanol as a blank. The optical density was then determined at 517 nm. To find the synthesized ZnO NPs' DPPH scavenging activity, the following equation (1) was applied:

(1) $$\%\;\text{of}\;\text{DPPH}\;\text{inhibition}=\left\{\left(A_{0}\;–\;A_{s}\right)/A_{0}\right\}\times 100$$

Here, control is A_0_ and sample is A_s_ to identify % of inhibition.

### 2.6 Antimicrobial acivity

#### 2.6.1 Test microorganisms

The research investigation examined the antibacterial activity of synthesized ZnO NPs against three strains of *S. aureus*, *E. coli*, and *P. aeruginosa* that are resistant to antibiotics. First, tainted food samples were used to extract the test bacteria. To create pure cultures, the isolated bacteria were regularly subcultured on Nutrient Agar (NA, Hi-media, India) and then incubated at 37 °C. The isolated pure cultures of bacteria were kept at −20 °C right away. The selected isolates were then identified by performing the series of morphological (growth on suitable media), microscopic, physiological, and biochemical tests. Using carbon sources and a chemical sensitivity test, the BIOLOG™ identification system was used to determine the species level [40].

#### 2.6.2 Kirby Bauer disc diffusion assay

Prior to the experiment, all of the glassware and associated items were autoclaved at 121 °C and a pressure of 115 lb. The modified Kirby Bauer disc diffusion method followed by the Clinical and Laboratory Institute (CLSI) guideline was utilized to assess the antibacterial susceptibility test [41]. Amikacin 30 μg disc was considered as a positive control whereas 10 % DMSO soaked filter paper disc was served as a negative control. ZnO-NPs (100 μg) were dissolved in 1.0 mL of 10 % DMSO. 100 μL of the prepared sample solution was applied on the antibiotic disc. The plates were subsequently kept at 4 °C for 3 h to allow nanoparticles to enter and diffuse across the well, after which incubation at 37 °C was performed. Clear zones were discovered surrounding the wells after 24 h of incubation, and a slide calliper was used to measure these zones' diameters.

#### 2.6.3 Minimum inhibitory concentrations (MIC) and minimum bactericidal concentrations (MBC)

The broth micro dilution method was employed to examine the MIC and MBC following the CLSI guideline [42]. Three concentrations doses of ZnO NPs: 50 μg, 100 μg and 200 μg in 10 % DMSO were investigated against the bacterial concentration of 5 × 10^5^ CFU/mL. Bacterial suspension (50 μL) was added to each sample except the negative control (10 % DMSO). Amikacin 30 (30 μg) was considered as a positive control. Both positive control and ZnO-NPs coated discs were incubated for 24 h at 37 ± 2 °C. After incubation, MIC and MBC was determined by visual inspection.

### 2.7 Statistical analysis

All the antioxidant and antibacterial experiments were carried out three times and the results are presented as mean ± standard deviation. We used R Studio (version 4.3.0, 2019), OriginPro (2022) and Microsoft Excel (Office 2016) to draw the graph. No test was performed to determine the level of significant difference (p < 0.05).

## 3 Results and discussion

### 3.1 UV–vis Spectroscopic analysisand synthesis method optimization

**Fig. 1:**
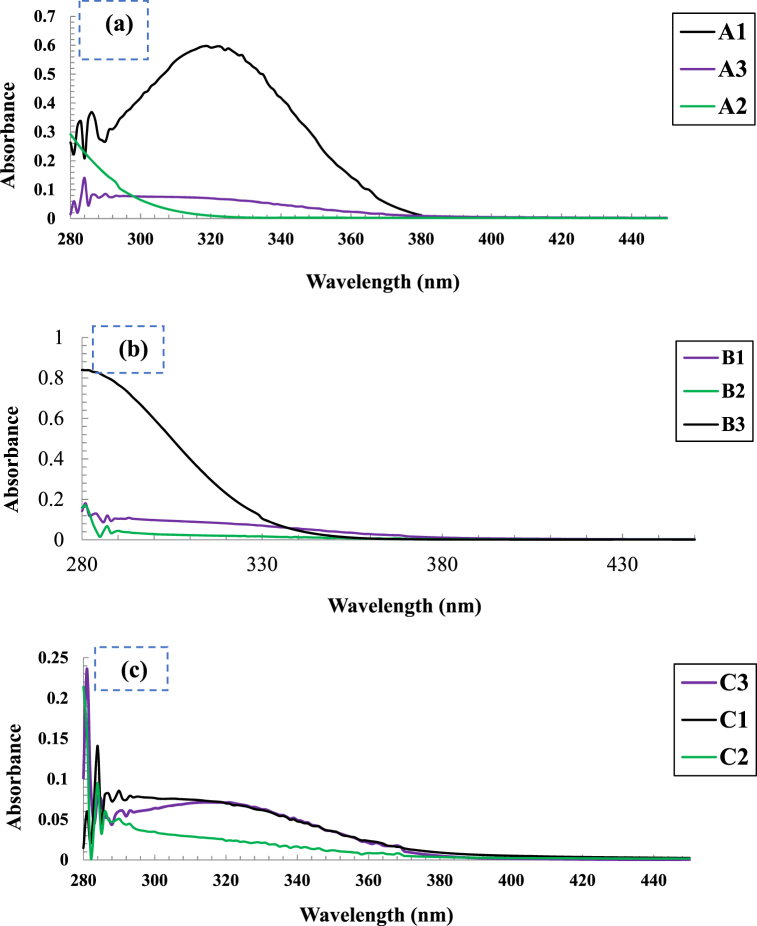
UV–Vis spectra of synthesized ZnO-NPs. **1(a)**: synthesized using extract from condition A, **1(b)**: synthesized using extract from condition B and **1(c)**: synthesized using extract from condition C.

The formation of ZnO-NP using secondary metabolites of onion waste peel were investigated by UV–visible spectra in the range of 280 nm–450 nm. As shown in Fig. 1(a), sample A1 exhibited an absorption maximum at 322 nm, which corresponds to the ZnO-NPs whereas A2 and A3 didn't exhibit any absorption maxima. A1 was synthesized from the most concentrated extract and this indicated that the microwave heat treatment was more effective when the substrate to solvent (w/v) ratio was maimtained 1:2 to prepare the extract solution. No absorption maxima were observed for condition B experiments shown in Fig. 1(b) and this implied that ZnO-NPs were not formed when the extract was prepared only by orbital shaking. The reason might be due to the incomplete extraction of the reducing agent. So, orbital shaking is not an efficient technologies to extract the capping and reducing agent from the dry onion waste peel to synthesize ZnO NPs. In case of condition C, the strongest peak was noted for sample C3 at 318 nm whereas C1 and C2 did not show any significant absorption maxima presented in Fig. 1(c). Since the samples A1 and C3 only exhibited intense UV–visible absorption maxima among the nine samples and based on this data these two samples were chosen for further characterization. Subsequently, evaluation of their antioxidant and antibacterial activities to make a comparative study in between the synthesized ZnO NPs utilizing extract solution prepared from two different approaches.

**Fig. 2:**
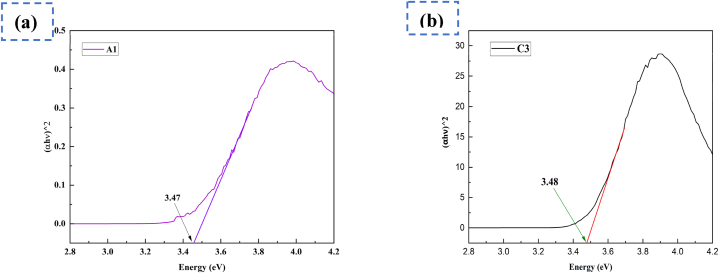
Tauc plots for optical bandgap energy. For A1 sample **(a)** and C3 sample **(b)**.

ZnO-NPs surface plasmon absorption properties are the cause of all the peaks [43]. These results matched with the absorption pattern of conventional ZnO since oxide materials have shorter wavelengths and broad band gaps [44,45]. The synthesized A1 and C3 samples estimated band gap energy shown in the Tauc plot in Fig. 2(a) and **2(b)** respectively.

Using the following equation (2), the band gap energy (E_g_) is calculated by extrapolating the linear part of (αhv)^2^ against energy plots [46].

(2) $$\alpha h\nu =A{\left(h\nu -E_{g}\right)}^{n}$$

where A is the constant, E_g_ is the optical band gap energy, hν is the photon energy, and α is the absorption coefficient. The value of n equals 1/2 in the case of permitted direct transitions, such as those in ZnO. The energy band gaps are indicated by the intercepts of these graphs on the energy axis, as Eg = hν when (αhν)^2^ = 0. The computed band gap value was found 3.47 and 3.48 eV respectively for our sample A1 and C3, and this value indicated that synthesized ZnO-NPs absorb UV light. A band gap of 3.32 eV is estimated for ZnO NPs synthesized via green synthesis [35]. So, it can be used for medicinal purposes like protection of skin from sun and/or antibacterial ointments [47]. The broad spectrum of energy band gap of our synthesized ZnO-NPs also indicates their possible use as metal oxide semiconductor-based systems such as memory devices, sensors, UV-light emitting diodes, photocatalysts, solar cells, photodetectors and piezo-electric transducers and is similar to the energy bandgap values that have been previously reported for ZnO-NPs [48].

### 3.2 FE-SEM characterization of ZnO NPs

**Fig. 3:**
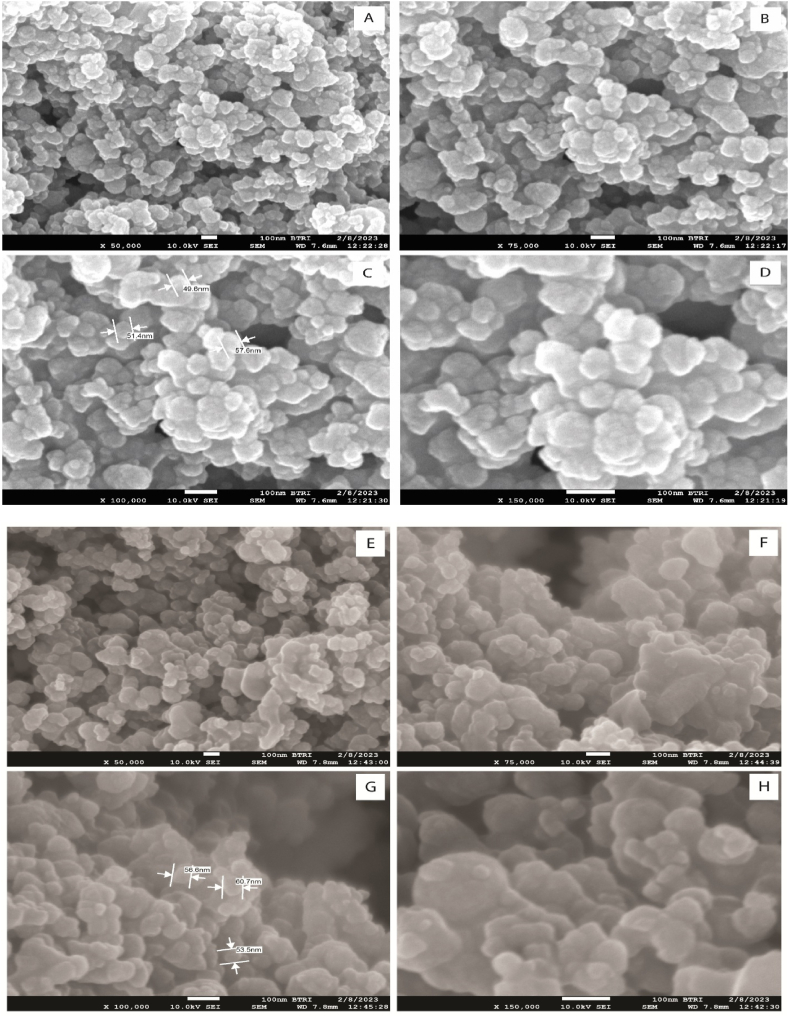
FE-SEM images of ZnO-NPs. **(A**–**D)** Forsample A1 and **(E**–**H)** for sample C3 captured at various magnifications. The scale denotes 100 nm.

The morphological study of synthesized ZnO-NP were performed using FE-SEM scanned at different magnification. Fig. 3(A-H) showed the morphological study of the green synthesized ZnO-NPs from samples A1 shown in Fig. 3 (A-D) and C3 presented in Fig. 3 (E-H). At low magnification (50000×), the FE-SEM images of A1 and C3 showed notable agglomeration, which point towards the distinct characteristics of ZnO-NPs fabrication mediated by plant extracts. At higher magnifications (100000× and 150000x), the image revealed their regularly clustered hexagonal-shaped nanoparticles for sample A1 that were heterogeneous in nature. The size of the agglomerated particles for sample A1 ranged from 49 nm to below 100 nm as shown in Fig. 3(C). The agglomeration of smaller particles leads to the creation of larger particles, which also gives irregular shapes to the structures [49]. The hexagonal morphology and agglomeration with irregular distribution pattern of the synthesized ZnO-NPs were evident from previous studies [50,51].

Fig. 3(G) shown the irregularly shaped nanoparticles with both round and hexagonal shapes, obtained from sample C3 using higher magnification levels (100,000× and 150,000x). These nanoparticles have sized ranged from 53 to 100 nm. The round shaped of ZnO-NPs has also been observed in a research study conducted by Ihsan et al. [52]. The increased of the particle size and distortion of the hexagonal structure to round shaped in sample C3 from sample A1 attributed to the increased concentration of the extract in sample A1 than C3 and this effect of concentration was consistent with the result reported in previous study [34]. ZnO-NPs formation was confirmed by further XRD analysis.

### 3.3 Energy Dispersive X-ray analysis (EDX)

**Fig. 4:**
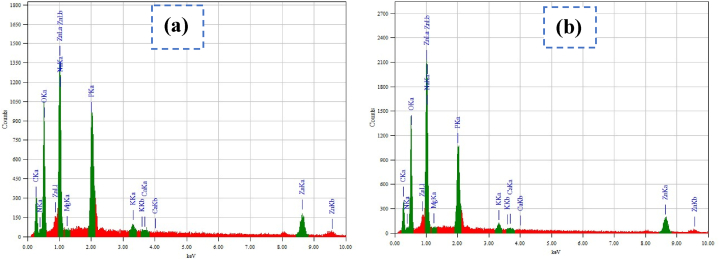
EDX spectrograph of bio-synthesized ZnO NPs. **(a)** For A1 and **(b)** C3 sample.

The EDX pattern of sample A1 and sample C3 showed that the single peak of O was found between 0 and 1 eV and three peaks for Zn were found at 1eV and between 8 and 10 eV shown in Fig. 4(a) and **4(b**) which confirmed the presence of zinc and oxygen in the oxide form. The EDX spectra of synthesized ZnO-NPs also confirmed that Zn and O comprised 37.23 % and 25.62 % of the mass of sample A1 whereas 33.45 % and 29.32 % of the mass of sample C3. Apart from Zn and O, the observed peak for C reflected the green feature of the nanoparticle synthesis process, and the mass percentage of C accounts for the stabilizing and capping of the green synthesized nanoparticles [53].

### 3.4 FT-IR analysis of ZnO-NPs

**Fig. 5:**
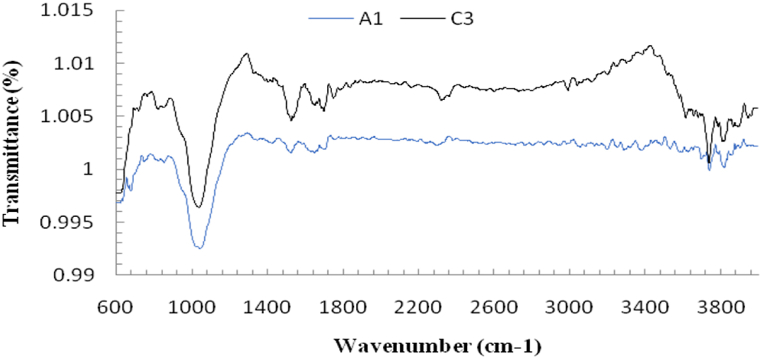
FTIR pattern of synthesized ZnO NPs from two different conditions.

The onion waste peel extract mediated synthesized ZnO NPs FTIR spectra were captured between wavenumber of 600–4000 cm^−1^ and presented in Fig. 5. Phytochemicals present in the onion waste peel extract could be involved in the capping and reduction of the Zn^2+^ ions. The FT-IR analysis of sample A1 exhibited bands at 2302.99 cm^−1^, 1697.96 cm^−1^, 1649.08 cm^−1^, 1524.91 cm^−1^, 1426.08 cm^−1^, 1045.16 cm^−1^, 747.03 cm^−1^, 664.40 cm^−1^, 634.40 cm^−1^ and 630.32 cm^−1^. The C=O double bond and C–O stretching with slight shift are indicated by a strong and medium peak at 1649.08 cm^−1^ and 1697.96 cm^−1^, respectively [54]. A strong band at 1524.91 cm^−1^ corresponds to the N–O stretching present in nitro compounds. Medium peak at 1426.08 cm^−1^ is attributed to O–H bending of alcohols. The sharp band at 1045.16 cm^−1^, 747.03 cm^−1^, and 664.40 cm^−1^ were attributed to the stretching of S–O, C–Cl, and C–Br, respectively. Two sharp bands at 634.40 cm^−1^ and 630.32 cm^−1^ are observed due to the interaction of ZnO-NPs with other organic moieties. The 500-900 cm^−1^ peaks are attributed to the metal-oxygen groups [55]. For sample C3, bands found at 2322.73 cm^−1^, 1783.99 cm^−1^, 1750.52 cm^−1^, 1525.34 cm^−1^, 1038.46 cm^−1^, 822.61 cm^−1^ and 665.13 cm^−1^. The medium peak at 2322.73 cm^−1^ is attributed to C=C alkenes. Two strong peaks at 1783.99 cm^−1^ and 1750.52 cm^−1^ correspond to C=O monomer. N–O stretching is attributed by a strong band at 1525.34 cm^−1^ and this stretching is of nitro compounds. Intense peak at 1038.46 cm^−1^ corresponds to S–O stretching of sulfoxide. ZnO intra-atomic weak interactions are indicated by a strong peak at 822.61 cm^−1^ and 665.13 cm^−1^ which is allotted for ZnO as reported by A. Jayachandran et al. [56].

The slight shift of FTIR spectra of ZnO-NPs could occurred for the different experimental conditions. The presence of C=C bonds, S–O stretching of sulfoxide, nitro compounds N–O stretching and C=O monomers reveals that these groups assist the reduction and capping of Zn^2+^ ions and the overall formation of ZnO-NPs and this is corroborated by previous studies [52,57]. The donor-acceptor mechanism was followed for the reduction of Zn^2+^ions in green synthesis. The presence of hydroxyl or oxygen compounds in the onion waste peel extract give an electron to an electrophile zinc complexes cause zinc ions to be reduced and OH groups to be oxidised [58]. The S–O stretching represents the abundance of organo-sulfur compounds in *Allium cepa* L [59]. Despite multiple washings, the FTIR data showed the existence of metabolites, such as flavonoids, alkaloids, carboxylic acid, and polyphenols, which chained to ZnO-NPs. These substances, in particular flavonoids and phenolics, helped transform zinc ions into ZnO-NPs. Apparently, the presence of carboxylic groups stabilizes ZnO-NPs by interacting with the Zn surface [60]. Therefore, FTIR data revealed that the presence of different functional group containing bioactive compouds in the onion waste peel extract made the synthesis procedure effective and formation of NPs successful.

### 3.5 XRD analysis

**Fig. 6:**
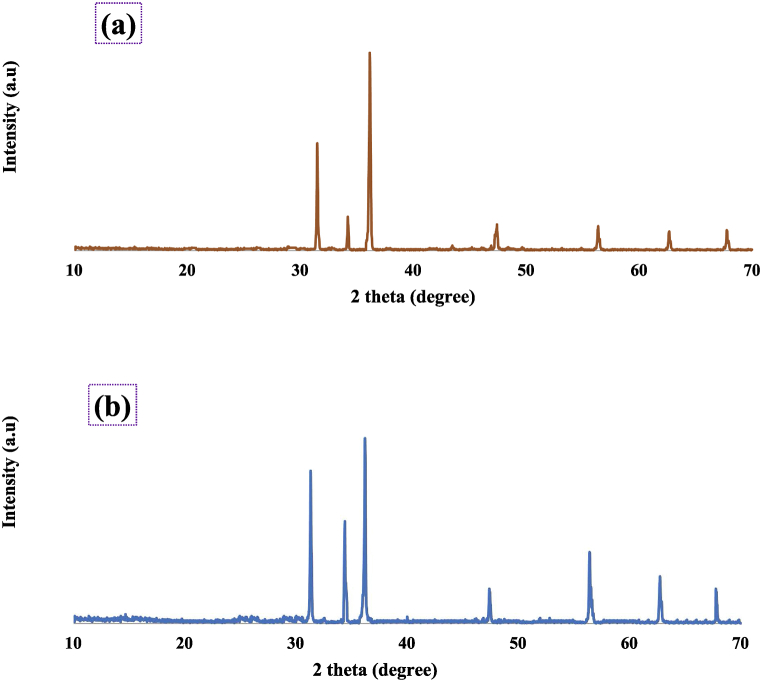
X-ray diffraction spectra of synthesized ZnO NPs for A1 (a) and C3 (b).

The XRD pattern of A1 and C3 sample is depicted in Fig. 6(a) and (b). The details of the crystal plane, nature, and size of the NPs provided by XRD [61]. For both sample A1 and C3, the diffraction peaks at lattice planes (hkl) indexed as (100), (002), (101), (102), (110), (103), and (112) have been used to identify the spherical to hexagonal phase of the produced ZnO-NPs. The crystal's peaks were found to match the peaks on the ICDD (International Centry for Diffraction Data) card no #01-079-0207 [57]. Line broadening in XRD peaks indicated the nanoscale range of the synthesized nano particles. Sharp peak also supported the crystalline nature of NPs [54].

The Debye-Scherrer formula (equation (3)) was employed to compute the diameter of ZnO crystals size.

(3) $$D=\frac{k\lambda }{\beta \;\cos\;\theta }Å$$

Here, D is the mean particle size of nonmaterials, k is the shape factor or Scherrer's constant (0.9), λ is the wavelength of x-ray (1.5406 Å), β is the full width at half maxima in radians and θ is the Bragg's diffraction angle in degrees. The more intense peaks corresponded to (101) planes and smallest crystal size was found for both samples. It revealed that most of the particles were pointed in (101) direction. The others diifraction peak at higher 2θ value might be responsible for the capping and stabilizing agent of onion waste peel extract species. The detail calulation of particle size data is provided in the supporting information titled as table S1. The average crystallite size was found 72.60 and 57.38 nm, respectively, for samples A1 and C3. The result was compatible with the findings of SEM. The particle size of nanomaterials plays crucial role in their different activities especially in the antioxidant and antibacterial efficacy.

### 3.6 Antioxidant activity

**Fig. 7:**
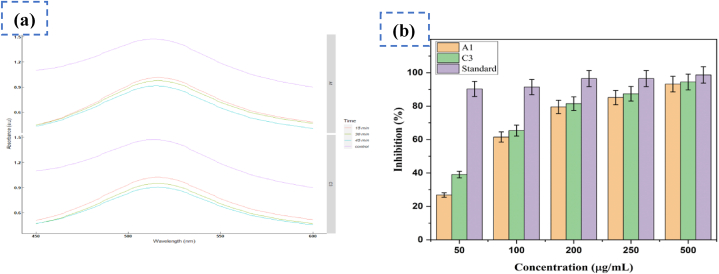
ZnO NPs antioxidant activity at various time intervals **(a)** and at different concentrations **(b).**

As seen in Fig. 7(a) and (b), the antioxidant activity of the produced ZnO NPs was assessed using the DPPH scavenging assay. For both samples shown in Fig. 7(a), the maximum intensity at 517 nm gradually reduced with time in agreement with changes in color of the DPPH solution in the presence of ZnO NPs. This finding correlated with the result observed by D. Das et al. in 2013 [39]. The drop in peak intensity proofed that ZnO nanoparticles capable to scavenge free radicals. The DPPH scavenging activity of sample A1 and C3 were found 63.85 % and 71.53 % at 45 minrespectively. The DPPH free radical scavenging activity data at different concentration has been provided in the supplementary documents **S3**.

The antioxidant activity of green synthesized ZnO-NPs was found higher when concentration was higher for both samples but lower than the standard. This results is accordant with the findings reported by Adamu AS S et al. [34]. Sample C3 showed increased free radical inhibition activity than A1 at every concentration. Therefore, it was revealed that DPPH free radical scevanging activity of ZnO NPs was dose dependent and NPs with smaller particle size exhibited enhanced antioxidant activity at the same concentration.

### 3.7 Antimicrobial activity

**Fig. 8:**
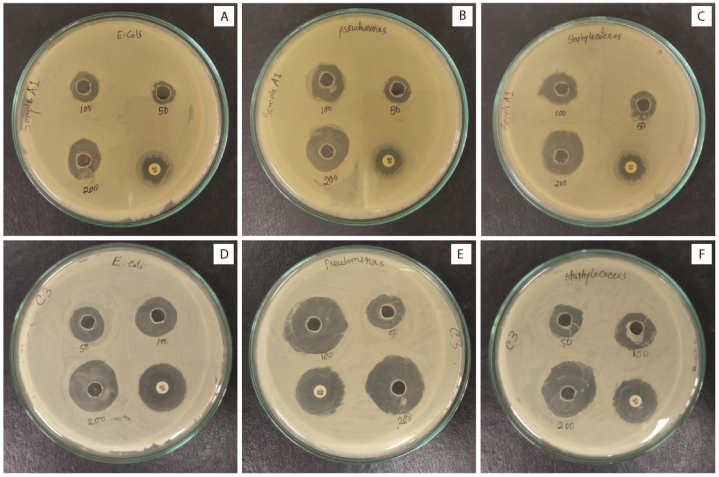
Antibacterial activity of ZnO-NPs against three bacterial strains at three different concentrations. **(A**–**C)** is for sample A1 and **(D**–**F)** is for sample C3.

The antimicrobial activities shown in Fig. 8 (A-F) of the synthesized complexes were studied *in-vitro* against 2 g-negative strains *P. aeruginosa* and *E. coli* and 1 g-positive strain *S. aureus* using disc diffusion method with reference antibiotic Amikacin 30 (30 μg disc). The results exhibited that the synthesized ZnO-NPs possessed a noteworthy antibacterial properties against all three tested microorganisms. It was shown that sample C3 showed significant zone of inhibition than sample A1 at all concentration. This behavior might be for the small particle size of sample C3 than A1. When the particle size decreased, surface area increased resultant in the proper diffusion of NPs in the medium. This causes an elevated rate of surface oxygen species formation to accelerate membrane rupture and ultimately cause pathogen death [62]. Another explanation for ZnO NPs' antibacterial action could be that they come into direct contact with pathogens' cell walls, breaking down their cell walls and releasing antimicrobial ions (Zn^2+^) as a result. These free ions then bind with the carbohydrates and proteins of the pathogens, resulting in the disintegration of every key bacterial activity [37]. In our study, 50, 100 and 200 μg/mL concentrations of A1 and C3 samples were used to record the antibacterial activity after 24 h of incubation. No labeled at the assay plate was for standard Amikacin 30. The average zone of inhibition calculation data of both samples at three different concentration is tabulated in the supplementary file **S4** in detail. At 100 μg/mL concentration, gram positive bacteria *S. aureus* showed average zone of inhibition diameter 12.00 ± 1.52 mm and 13.50 ± 0.76 mm for samples A1 and C3 respectively. But, the scenario was quite different when dealing with gram negative bacteria *E. Coli* and *P. aeruginosa*. It is showed that sample A1 and C3 respectively cleared 8.83 ± 0.54 mm and 13.17 ± 0.82 mm diameter zone against *E. Coli* whereas 13.00 ± 1.00 mm and 22.00 ± 0.58 mm diameter zone was inhibited when studied against *P. aeruginosa*. The lowest zone of inhibition diameter 8.83 ± 0.54 mm was found for sample A1 against gram-negative *E. coli* bacteria whereas highest zone of inhibition 22.00 ± 0.58 mm was found for sample C3 against gram-negative *P. aeruginosa* bacteria at 100 μg/mL. Also, it was observed that for both sample A1 and C3, the highest zone of inhibition was found against *P. aeruginosa* and *S. aureus*. Among the three studied starin, *E. coli* was less sensitive i.e., sample A1 and C3 did not inhibit the significant zone when compared with the standard. However, between the studied sample, C3 exhibited highest zone of inhibition at all three concentrations, which indicated that the antibacterial activity was directly related to the particle size of ZnO-NPs [63]. In line with the earlier research, it was therefore demonstrated that as small as the particle size, the higher the antibacterial activity [62].

### 3.8 Minimum inhibitory and bactericidal concentration

The minimum bactericidal concentration (MBC) was the lowest amount of an antibacterial agent required to completely eradicate a particular bacterium, while the minimum inhibitory concentration (MIC) was considered the lowest amount of the materials (ZnO-NP) that completely prevented bacterial growth. The data was displayed in Table 1. It was observed that at 100 μg/mL, ZnO NPs were effective against three studied bacterial strains. The synthesized ZnO NPs were found to be 100 μg/mL and 200 μg/mL of MIC and MBC, respectively, for all selected strains. Therefore, ZnO-NPs synthesized with *Allium cepa* L. waste peel extact could be suggested as a good antibacterial material.

**Table 1:** Antibacterial activity of assynthesizedZnO nanoparticles against test microorganisms.

Sample A1			
Concentration (μg/mL)	Bacterial growth		
	S. aureus	E. coli	P. aeruginosa
50	–	+	–
100	–	–	–
200	–	–	–
MIC	50 μg/mL	100 μg/mL	50 μg/mL
MBC	100 μg/mL	200 μg/mL	100 μg/mL
Sample C3			
50	–	–	–
100	–	–	–
200	–	–	–
MIC	50 μg/mL	50 μg/mL	50 μg/mL
MBC	100 μg/mL	100 μg/mL	100 μg/mL

Note:‘+’ indicates the presence of bacterial growth; ‘-’ indicates no bacterial growth. MIC = minimum inhibitory concentration and MBC = minimum bactericidal concentration.

### 3.9 Comparative study

Numerous researchers have synthesized ZnO NPs via biogenic approach during the last two decades and examined their antioxidant and antibacterial properties and have demonstrated the NPs' strong antibacterial activity. We have synthesized ZnO NPs via green system from two different pathway and evaluated their structural and biological activity to explore its potentiality as an antioxidant and antibacterial materials. The elemental composition and biological activities data summary of our studied materials was presented in Table 2 to make the comparision easier between these two samples. Based on the analyzed data it could be concluded that C3 NPs were superior than the A1 NPs considering the antioxidant and antibacterial activities.

**Table 2:** Elemental composition and biological activity data of the synthesized two ZnO NPs.

Characterization parameters	ZnO NPs (A1)	ZnO NPs (C3)
**Absorption maxima**	322 nm	318 nm
**Estimated optical band gap calculated from Tauc plot**	3.47 eV	3.48 eV
**Morphology by FE-SEM**	Regularly clustered hexagonal-shaped with 49–100 nm	Regularly clustered hexagonal-shaped with 49–100 nm
**Average particle size calculated from Scherrer formula**	72.60 nm	57.38 nm
**Antioxidant activity (at 100 μg/mL)**	61.53 %	65.39 %
**Antibacterial activity (MZI)**	.	.
***S. aureus***	12.00 ± 1.52 mm	13.50 ± 0.76 mm
***E. coli***	8.83 ± 0.54 mm	13.17 ± 0.82 mm
***P. aeruginosa***	13.00 ± 1.00 mm	22.00 ± 0.58 mm
**Antibacterial activity (MIC)**	.	.
***S. aureus***	50 μg/mL	50 μg/mL
***E. coli***	100 μg/mL	50 μg/mL
***P. aeruginosa***	50 μg/mL	50 μg/mL
**Antibacterial activity (MBC)**	.	.
***S. aureus***	100 μg/mL	100 μg/mL
***E. coli***	200 μg/mL	100 μg/mL
***P. aeruginosa***	100 μg/mL	100 μg/mL

*MZI = maximum zone of inhibition at 100 μg/mL, MIC = minimum inhibitory concentration, and MBC = minimum bactericidal concentration.

## 4 Conclusion

ZnO-NP was synthesized successfully using aqueous *Allium cepa* L. waste peel extract through the optimization of extraction and synthesis method. The procedure was economical and environmentally friendly since it used extract from onion waste peels. The characterization was conducted via different techniques like UV–vis, XRD, FTIR, and FE-SEM. The average crystal size of the best-studied ZnO-NP was measured 57.38 nm with hexagonal shape. In our study, we have shown the possible bioactivity of green synthesized ZnO NPs by studying the antioxidant and antibacterial activity using standard method. When compared to the standard antibacterial substance, the growth of inhibition in synthesized ZnO NPs was found exclusively greater. Also, ZnO NPs and standard exhibited the similar antioxidant activity at higer concentration. The larger surface to volume ratio of smaller particles is thought to be responsible for their improved bioactivity. The bioresource-based ZnO NPs' exceptional bactericidal efficacy against gram (+)ve and gram (−)ve bacterial strains may lead to new opportunities for its application in food and medicine for the development of prospective antimicrobial drugs. However, *in vivo* research is necessary to fully comprehend their biological activities and may be the focus of future studies.

## Data availability

Data will be made available on request.

## CRediT authorship contribution statement

**Md Faridul Islam:** Supervision, Resources, Project administration, Investigation. **Shariful islam:** Writing – review & editing, Writing – original draft, Visualization, Investigation, Data curation. **Md Abdus Satter Miah:** Writing – review & editing, Project administration, Funding acquisition. **A.K. Obidul Huq:** Supervision, Conceptualization. **Anik Kumar Saha:** Methodology, Investigation, Formal analysis. **Zinia Jannat Mou:** Formal analysis. **Md Mahmudul Hassan Mondol:** Writing – review & editing. **Mohammad Nazrul Islam Bhuiyan:** Writing – review & editing, Resources, Methodology.

## Declaration of competing interest

The authors declare that they have no known competing financial interests or personal relationships that could have appeared to influence the work reported in this paper.
