# Supplementary material for: Green synthesis of zinc oxide nano particles using Allium cepa L. waste peel extracts and its antioxidant and antibacterial activities
Source: Heliyon. 2024 Jan 27;10(3):e25430. doi: 10.1016/j.heliyon.2024.e25430 (PMC10850583; doi:10.1016/j.heliyon.2024.e25430)
Supplement: Multimedia component 1 [file mmc1.docx]

**Tab.S1:** Crystal planes and average particle size details from the XRD analysis of ZnO-NPs synthesized at different diffraction angles

| **2 theta** | | **FWHM (β)** | | **Miller indices** | | **Particle size (nm)** | | **Average particle size (nm)** | |
| --- | --- | --- | --- | --- | --- | --- | --- | --- | --- |
| **A1** | **C3** | **A1** | **C3** | **A1** | **C3** | **A1** | **C3** | **A1** | **C3** |
| 31.34 | 31.52 | 0.00187 | 0.00271 | (100) | (100) | 77.01 | 53.16 | 72.60 | 57.38 |
| 34.42 | 34.22 | 0.00173 | 0.00252 | (002) | (002) | 83.91 | 57.57 |  |  |
| 36.22 | 36.16 | 0.00313 | 0.00328 | (101) | (101) | 46.61 | 44.47 |  |  |
| 47.40 | 47.42 | 0.00283 | 0.00324 | (102) | (102) | 53.50 | 46.74 |  |  |
| 56.42 | 56.38 | 0.00202 | 0.00244 | (110) | (110) | 77.89 | 64.47 |  |  |
| 62.76 | 62.68 | 0.00202 | 0.00279 | (103) | (103) | 80.40 | 58.19 |  |  |
| 67.78 | 67.76 | 0.00188 | 0.00314 | (112) | (112) | 88.85 | 53.19 |  |  |
